# Effects of Resistance Training and *Bowdichia virgilioides* Hydroethanolic Extract on Oxidative Stress Markers in Rats Submitted to Peripheral Nerve Injury

**DOI:** 10.3390/antiox9100941

**Published:** 2020-10-01

**Authors:** Luana Santos Costa, Felipe J. Aidar, Dihogo Gama de Matos, José Uilien de Oliveira, Jymmys Lopes dos Santos, Paulo Francisco de Almeida-Neto, Raphael Fabrício de Souza, Danielle Dutra Pereira, Nuno Domingos Garrido, Albená Nunes-Silva, Anderson Carlos Marçal, Charles dos Santos Estevam, Breno Guilherme de Araújo Tinoco Cabral, Victor Machado Reis, Mauro Martins Teixeira

**Affiliations:** 1 Program of Physiological Science, Federal University of Sergipe (UFS), São Cristovão 49100-000, Sergipe, Brazil; luanaenfermagem@academico.ufs.br; 2 Group of Studies and Research of Performance, Sport, Health and Paralympic Sports (GEPEPS), Federal University of Sergipe (UFS), São Cristovão 49100-000, Sergipe, Brazil; dihogo.dematos@mail.mcgill.ca (D.G.d.M.); uilien.oliveira@uilien.edu (J.U.d.O.); jymmys@academico.ufs.br (J.L.d.S.); desouza@ufs.br (R.F.d.S.); acmarcal@academico.ufs.br (A.C.M.); 3 Department of Physical Education, Federal University of Sergipe (UFS), São Cristovão 49100-000, Sergipe, Brazil; 4 Program of Physical Education, Federal University of Sergipe (UFS), São Cristovão 49100-000, Sergipe, Brazil; 5 Program in Biotechnology, Northeast Network in Biotechnology (RENORBIO), Federal University of Sergipe, São Cristovão 49100-000, Sergipe, Brazil; cse@ufs.br; 6 Department of Physical Education, Federal University of Rio Grande do Norte, Natal 59078-970, Brazil; paulojitte@ufrn.edu.br (P.F.d.A.-N.); brenotcabral@reitoria.ufrn.br (B.G.d.A.T.C.); 7 Department of Physiology and Pharmacology, Center of Biosciences, Federal University of Pernambuco, Recife 50670-901, Brazil; danielle.dutra@ufpe.br; 8 Health Sciences and Human Development (CIDESD), Research Center in Sports Sciences, University of Trás-os-Montes and Alto Douro, 5001-801 Vila Real, Portugal; ngarrido@utad.pt (N.D.G.); vmreis@utad.pt (V.M.R.); 9 Laboratory of Inflammation and Exercise Immunology, Physical Education School, Federal University of Ouro Preto, Minas Gerais 35400-000, Brazil; albena.silva@ufop.edu.br; 10 Department of Pathology, Institute of Biological Sciences, Federal University of Minas Gerais, Belo Horizonte 31270-901, Brazil; mmtex@ufmg.br

**Keywords:** peripheral nerve injury, resistance training, oxidative stress, skeletal muscle

## Abstract

The objective of this study was to analyze the effects of the combination of resistance training (RT) and the hydroethanolic extract (EHE) of *Bowdichia virgilioides* as markers of oxidative stress (OS) in rats with peripheral nerve injury (PNI). Rats were allocated into six groups (*n* = 10): animals without interventions (C), animals with an exposed nerve but without injury, injured animals, trained and injured animals, injured animals that received EHE, and animals that received a combination of RT and EHE. RT comprised the climbing of stairs. EHE was orally administered (200 mg/kg) for 21 days after PNI induction. RT reduced the amount of lipoperoxidation in plasma (14.11%). EHE reduced lipoperoxidation in the plasma (20.72%) and the brain (41.36). RT associated with the extract simultaneously reduced lipoperoxidation in the plasma (34.23%), muscle (25.13%), and brain (43.98%). There was an increase in total sulhydrilyl levels (a) in the brain (33.33%) via RT; (b) in the brain (44.44%) and muscle (44.51%) using EHE; and (c) in the plasma (54.02%), brain (54.25%), and muscle using the combination of RT + EHE. These results suggest that RT associated with oral EHE results in a decrease in OS.

## 1. Introduction

Peripheral nerve injury (PNI) is a neurological disorder that affects approximately 2–3% of victims of polytrauma; the largest affected audience is young men [1,2,3]. However, its incidence is still often underestimated, and there is a lack of clinical studies pointing toward important epidemiological data of the pathology [4].

Nerve damage is associated with sensory deficits, pain, and impaired skeletal muscle function, which can result in tissue damage, loss of muscle mass, decreased motor function, and disability [5,6,7]. In addition to causing physical changes, PNI is related to psychological, behavioral, and social impacts [8,9]. The most prominent causes are car accidents, firearm and stab injuries, falls, autoimmune diseases, infections, metabolic disorders, and iatrogenic causes [10,11].

Depending on the extent of the damage, PNI can be categorized into different degrees: (1) neuropraxia: mild neuronal damage, commonly induced by ischemia or stretching; in this case, the nerve is intact but does not transmit impulses; (2) axonotmesis: intermediate lesions in which the axon ruptures but the surrounding connective tissue is intact; it is usually associated with nerve compression; and (3) neurotmesis: corresponding to the most severe degree of injury, in which there is total nerve rupture caused by section injuries [12,13]. These different types of injuries are related to different therapeutic applications, including spontaneous nerve recovery, the need for drug therapies, rehabilitation, and surgical repair in the most severe cases [14].

Damage to nerve endings promoted by PNI leads to the disuse of the skeletal muscle innervated by the injured branch [15]. Therefore, ischemic and inflammatory processes are involved, resulting in the emergence of oxidative stress (OS) [15,16]. Once the peripheral nerve suffers damage, there is accumulation of pro-oxidant substances; simultaneously, the levels of endogenous antioxidants are insufficient to maintain resistance to oxidative damage [15].

This increase in the levels of pro-oxidizing agents is capable of causing rupture of membranes via lipoperoxidation, rupture of deoxyribonucleic acid (DNA) chains, and changes in the structure or function of proteins, ultimately resulting in cell apoptosis [17]. OS in PNI, in addition to the involvement of the organ innervated by the injured nervous branch, covers the brain and plasma content, affecting several neural networks, blood components, and muscle metabolism [18].

At present, although there are several treatment types for PNI, most of them are ineffective in achieving functional recovery [19]. Therefore, alternative treatments have been studied to provide better regeneration process and functional improvement [20]. Physical exercise is recognized for offering a wide range of benefits and is well-documented as a form of therapy in several diseases for providing improvements in cardiovascular function, increased basal metabolism, psychosocial well-being, hyperphoria stimulation, and greater motor function, resulting in improved functional capacity to perform day-to-day activities [21,22].

Resistance training (RT) is a type of physical exercise that is well-acknowledged for its contribution in preventing and treating various disorders [23]. It also has a protective effect on PNI, although the mechanisms involved are unclear [24]. Continuous RT is capable of giving rise to physiological adaptations that can attenuate OS via the production of endogenous antioxidant system-activating mediators [23]. In particular, in PNI, RT has a protective effect because it is one of the most efficient methods to increase strength and muscle mass [24].

Another alternative strategy that has been studied in PNI is the use of natural products for their beneficial effects, such as improved motor performance, sensory function, and possible OS attenuation [25]. The bark and seeds of *Bowdichia virgilioides*, popularly known as sucupira-preta in Brazil, are widely used in folk medicine to treat various pathologies [26]. Our group has demonstrated the strong antioxidant effect of the hydroethanolic extract (EHE) of the stem bark of this tree both in vitro and in vivo [27]. In addition, its concomitant use with RT promotes a greater antioxidant effect than when used alone [27].

Therefore, considering the need for new therapies for PNI and its complications, we aimed to analyze the indicators of OS and muscle damage in aPNI rat model that was subjected to RT and oral EHE obtained from the stem bark of *B. virgilioides*.

## 2. Methods

This was a preclinical, experimental study conducted at the Research Center for Intracellular Signaling (NUPESIN), located in the Morphology Department of the Federal University of Sergipe (UFS), São Cristóvão, Brazil.

In total, 60 adult male Wistar rats (age: 90 days; body weight: 250–350 g) obtained from the NUPESIN Sectorial Animal Hospital were used. The rats were housed in cages kept at the vivarium under standard conditions of room temperature (22–24 °C) and light/dark cycle (12-h light/dark). Water and food were provided ad libitum. The experiment was conducted in accordance with the guidelines of the National Council for the Control of Animal Experimentation. The study was approved by the Ethics Committee on Animal Research, UFS (No. 02/2019, 1 March 2019).

Animals were randomly divided into six groups (each group comprising 10 animals): control group (C; sedentary animals that received a vehicle (distilled water 200 mg/kg, orally) and did not suffer any type of injury), sham group (S; sedentary animals that received a vehicle and in whom the right sciatic nerve was exposed but not injured), injury group (L; sedentary animals that received a vehicle and suffered an injury to the right sciatic nerve (preceded by the anesthetic plan)), injury + RT group (LT; animals that suffered injuries to the right sciatic nerve and were submitted to an exercise protocol and received a vehicle); injury + extract group (LE; animals that suffered lesions on the right sciatic nerve (preceded by the anesthetic plan] and were sedentary and received EHE [200 mg/kg, orally)), and injury + RT + EHE group (LTE; animals that suffered injury to the right sciatic nerve (preceded by the anesthetic plan) and received both an exercise training protocol and oral EHE at 200 mg/kg).

### 2.1. PNI Induction

**Figure 1:**
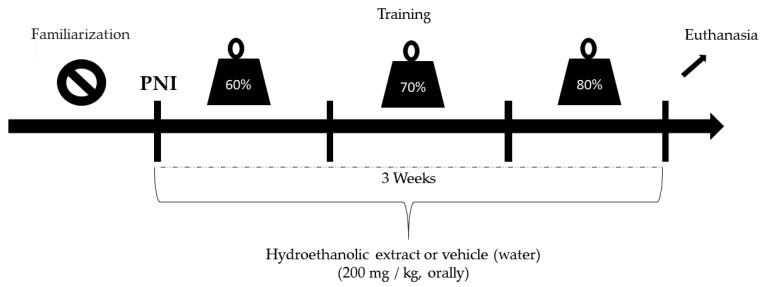
Study design.

The most commonly affected peripheral nerve is the sciatic nerve. In addition, it is the primary nerve preferred in rat experimental studies because it can be easily accessed surgically and contains well-located branches [10]. Therefore, after confirming the anesthetic effect (intra peritoneal ketamine (10 mg/kg) and xylazine (85 mg/kg) administration) by checking the state of consciousness and reflex to tail pinching, limb trichotomy was performed at the lower right region. First, asepsis of the region was performed using povidoneiodine, followed by an incision parallel to the fibers of the femoral biceps to expose the sciatic nerve. Next, the animals were subjected to unilateral crushing of the right nerve at the level of its trifurcation with the aid of surgical forceps for 30 s to trigger axonotmesis [28]. The same crushing pressure was applied for all animals. Postoperative care involved antibiotic prophylactic therapy with intraperitoneal administration of a single dose (0.1 mg/100 g) of pentabiotic and analgesia and opioid morphine (0.3 mg/100 g). The animals were then housed in air-conditioned cages (two rats per cage) containing water and feed. On day 2 after the injury, the animals were submitted to experimental protocols (Figure 1). The study design is illustrated in Figure 1.

### 2.2. Application of Therapeutic Resources

The therapy comprised the administration of EHE, RT, or both.

### 2.3. RT

For RT, a vertical wooden ladder (1.1 × 0.18 cm^2^, with 2 cm of space between the steps and an inclination of 80°) was used. Before initiating RT, the animals were first familiarized with the equipment for two weeks prior to PNI induction. Initially, the rats were motivated to climb the ladder by means of a pressure stimulus applied to the caudal end of the animal with metallic forceps. At the top of the stairs, a dark box (20 × 20 × 20 cm^3^) allowed the animals to rest between the series for approximately 2 min. This procedure was repeated until the rats voluntarily climbed the ladder three consecutive times [29].

After familiarization, the maximum repetition (1 RM) was determined, in which the animals climbed the ladder carrying progressively heavier loads. The initial load consisted of 50% of the animal’s body weight, with an increase of 10% each time until the animals reached 100% efficacy. The load for each training session started with 60% of that obtained in the 1 RM test and increased by 10% each week. Three sets of eight repetitions were performed.

The animals climbed the ladder carrying the load fixed to the proximal portion of their tail. RT was performed three times a week, on alternate days, for three weeks after the injury. The animals were weighed at before each training to adjust the load. Sedentary rats did not perform the exercise protocol but were placed in the training environment so that the stress of the environment was equal in all groups.

### 2.4. Collection and Extraction of the Barks of B.virgilioides

*B. virgilioides* barks were collected from Fazenda Riachão, Japaratuba, Sergipe, Brazil (10°32′04.49 S, 36°53′57″O). The desiccata of this species can be found in the herbarium of the Federal University of Sergipe, Brazil (ASE record 23, 107 [SISGEN AA5B37]). First, the barks were dried at room temperature and powdered using a knife mill before subjecting them to maceration with 90% ethanol for 5 days. Next, the solution was filtered and concentrated in an evaporator (LS LOGEN, London, UK) under reduced pressure at 45 °C, to obtain EHE.

EHE was administered via intragastric gavageat a dose of 200 mg/kg three times a week on 20 alternate days for three weeks [27] after the injury using a rodent-specific stainless-steel cannula with a rounded end. The control group animals received water (vehicle).

### 2.5. Phytochemical Prospecting

To detect the chemical components present in EHE and its fractions, phytochemical screening was performed according to the methods described by Matos [30] and our previous study [27].

The high-performance liquid chromatography system (HPLC) used contained a Prominence series liquid chromatograph (Shimadzu, Barueri, São Paulo, Brazil) with the C18 analytical column (25.0 × 0.46 cm, 5 μm particles) and a photodiode array detector. The LC Solution software was used to acquire and process the chromatographic data. EHE was analyzed using HPLC with an exploratory water: methanol gradient (5–100%) over 60 min to investigate the chromatographic profile of the phytochemicals. For this purpose, 2 mg of the EHE extract of *B. virgilioides* was dissolved in methanol to obtain a final concentration of 1 mgmL^−1^, which was filtered using 0.45-μm nylon membranes (2.5 cm d.i). Then, this sample was injected into the analytical HPLC ata flow of 1 mLmin^−1^. Detection was performed at 280 nm.

The presence of phenols and tannins was qualitatively analyzed via Folin’s reaction and FeCl_3_ reaction, respectively. Flavonoids, leucoanthocyanidins, catechins, and xanthones were analyzed via pH variation reactions. Steroids and triterpenoids were analyzed via the Lieberman–Burchard reaction. Saponins were analyzed via the formation of precipitates and foam. Alkaloids were detected via precipitation with Hager, Mayer, and Dragendorff reagents.

Phytochemical prospecting revealed the presence of secondary metabolites in the extract and fractions of *B. virgilioides* (Table 1).

### 2.6. Quantification of Total Phenol Content (PC)

Total PC was determined using the Folin–Ciocalteu reagent according to the methodology of Sousa et al. [31], with some modifications. EHE and its fractions (10 mg) were dissolved in 10 mL of methanol. An aliquot (100 µL) of the resulting solution was transferred to a falcon tube together with 6 mL distilled water and 500 µL Folin–Ciocalteu reagent (1 N). The solution was stirred for 1 min. After adding 2 mL of 15% Na_2_CO_3_, the mixture was stirred for 30 s. The solution was then diluted with distilled water to a final volume of 10 mL and incubated for 120 min at 23 °C. The absorbance of the samples was measured using an UV-VIS spectrophotometer model SP22 (Max Labor, São Paulo, Brazil) at a wavelength of 750 nm against white using quartz cuvettes. The PC content was determined by interpolating the absorbance of the samples against a calibration curve using gallic acid as a standard. The results were expressed as mg of gallic acid (GA) per g of extract or fraction. All analyzes were performed in triplicate.

### 2.7. Total Phenols

It was observed that the EHE extract showed a good concentration of phenolic compounds and subsequently a large number of hydroxyls. This result indicates that EHE has good biological activity, such as antioxidant activity. Spectrophotometric analysis revealed the PC content of EGEwas 128.05 ± 26.10 mg eq AG/g of extract.

### 2.8. DPPH Free Radical Scavenging Activity

The samples were dissolved in methanol to obtain a stock solution of 0.5 mg mL^−1^, from which aliquots were removed and added to a solution of 40 μg mL^−1^ 2,2-diphenyl-1-picrylhydrazyl (DPPH^•^) to obtain final concentrations of 5, 10, 15, 20, 25, and 30 μg mL^−1^ in a final reaction volume of 3 mL. The blank contained a mixture of the sample analyzed and methanol, with GA as the positive control.

The DPPH^•^ calibration curve was constructed based on the absorbance values at 515 nm, measured in a UV-VIS spectrophotometer model SP22 (Max Labor, São Paulo, São Paulo, Brazil) using quartz cuvettes. Absorbance measurements were performed at 1, 5, and 10 min and then at every 10-min interval until 60 min of incubation [31]. With the equation of the calibration curve and the absorbance values, in the time of 60 min for each concentration tested, the percentage of remaining DPPH (% DPPH_REM_) was determined and was calculated according to the equation by Brand-Willams et al. [32]:

%DPPH_REM_ = [DPPH]_T_/[DPPH]_T0_ × 100 (1)

where [DPPH]_T_ is the concentration of the radical in the reaction medium after the reaction with the sample and [DPPH]_T0_ is the initial concentration of DPPH.

The effective concentration of the antioxidant, required to decrease the initial concentration of the DPPH radical by 50% (EC_50_) was calculated using % DPPH_REM_ over 60 min compared with the concentrations of the samples. The results were expressed in μg mL^−1^ ± standard deviation (SD).

The absorbances measured at a concentration of 30 μgmL^−1^ L and over 60 min were transformed into percentage of inhibition (PI). Antioxidant activity was also expressed in terms of antioxidant activity index (AAI), which was calculated according to the equation by Scherer and Godoy [33]:

AAI = DPPH stock (μg mL^−1^)/EC_50_ (μg mL^−1^) (2)

The antioxidant activity was considered as weak when the AAI value was less than 0.5, moderate when the AAI was between 0.5 and 1.0, strong when the AAI was between 1.0 and 2.0, and very strong when the AAI value was greater than 2.0.

### 2.9. Euthanasia and Collection of Biological Material

On postoperative day 22, the animals were weighed and anesthetized via the intra peritoneal injection of ketamine (10 mg/kg) and xylazine (85 mg/kg). Next, under the influence of the anesthetic, the animals were euthanized by beheading using a guillotine. Subsequently, the brain, right gastrocnemius muscle, liver, and blood were extracted for further analysis. The collected blood sample was immediately centrifuged at 4000× *g* for 15 min at ± 4 °C. The supernatant was stored at −80 °C. The other organs were washed three times with 1.15% potassium chloride (KCl) solution, dried, and weighed. Thereafter, they were homogenized wherein each gram of the tissue was mixed with 5 mL KCl + 10 µL phenylmethyl sulfonyl fluoride (100 mmol/L) + 15 µL 10% Triton solution and then centrifuged at 3000× *g* for 10 min at −80 °C.

### 2.10. Oxidative Stress (OS) Analysis

#### 2.10.1. Determination of Malondialdehyde/Thiobarbituric Acid (TBARS) Reactive Substances In Vivo

Following the method described by Lapenna et al. [34], lipid oxidation was determined by measuring TBARS. Aliquots of 200 µL of the samples (blood and tissues) were added to a 400 µL solution containing trichloroacetic acid (TCA; 15%), HCl (0.25 N), TBA (0.375%), butylated hydroxytoluene (BHT; 2.5 mM), and 40 µL sodium dodecyl sulfate (8.1%) and were heated for 30 min at 95°C in an oven. The pH of the solution was adjusted to 0.9 with concentrated HCl. To prevent lipid peroxidation during heating, BHT was used. After cooling the solution to room temperature, 4 mL butanol was added, followed by centrifugation at 800 × *g* for 15 min at ± 4 °C. Next, the absorbance of the supernatant was measured at 532 nm. A molar extinction coefficient of 1.54 × 10^5^/M/cm was used. The TBARS results are expressed malondialdehyde (MDA) equivalents (nmol MDA eq/mL) for plasma and tissue samples.

#### 2.10.2. Determination of Total Sulfhydryls (Thiols)

Antioxidant activity in the plasma and tissues was quantified by determining the sulfhydryl (SH) groups according to the methodology described by Faure and Lafond [35]. Briefly, 50 μL aliquots of the samples were mixed with1 mL tris-EDTA buffer (pH 8.2). The absorbance (A1) was measured at 412 nm. The samples were then transferred to test tubes containing 20 μL DTNB (10 mM), diluted in methanol (4 mg/mL), and left undisturbed in a dark room. After 15 min, the absorbance (A2) was measured again. The SH concentration was calculated using the following equation: (A2 − A1) − B × 1.57 mM × 1000, and the result was expressed in nmol/mg tissue.

### 2.11. Statistical Analyses

Descriptive statistics were performed using measures of central tendency, mean (X) ± SD, and 95% confidence interval (CI). To verify the normality of the variables, the Shapiro-Wilk test was used considering the small sample size. To assess the performance between the groups, the one- or two-way ANOVA test was used with Bonferroni post-hoc correction. A *p* value of < 0.05 was considered statistically significant. All statistical analyses were performed using the Graph Pad Prism version 7.0 statistical program (GraphPad Software, San Diego, CA, USA).

## 3. Results

Free DPPH^•^ radical scavenging activity analysis revealed that EHE demonstrated an excellent antioxidant profile, suggesting the presence of effective chemical compounds to reduce DPPH^•^ by 50% (EC_50_) compared with GA, as well as the highest PI and amount of free radicals and higher levels of antioxidant activity (phytochemical prospecting and total phenols) (Table 2).

**Figure 2:**
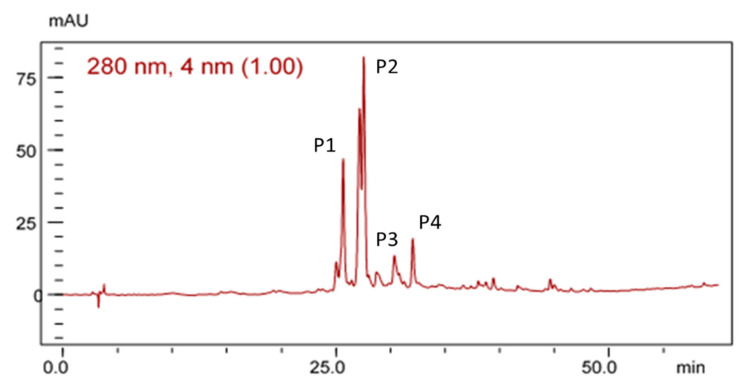
Chromatographic profile of the electrohyroalcoholic extract (EHE) of *B. virgilioides* under the conditions of exploratory gradient water/methanol (5–100%) in a 280-nm wave, Dos Santos [27].

Figure 2 shows the HPLC chromatographic profile of the EHE of *B. virgilioides*. The chromatogram demonstrated a characteristic fingerprint of medium- to high-polarity substances similar to phenolic compounds.

**Figure 3:**
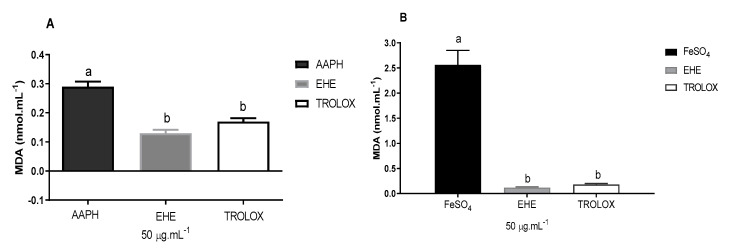
Effects of the hydroethanolic extract of *B. virgilioides* on the concentration of malondialdehyde (MDA) formed by the lipid peroxidation induced by AAPH (**A**) and FeSO_4_ (**B**). Results are presented as concentration of malondialdehyde formed (nmol mL^−1^). Values are expressed as means ± standard error of the mean. The letters “a” and “b” when repeated indicate statistical differences between the groups (*n* = 3). Statistical analysis was determined by via one-way ANOVA (Bonferroni post-hoc test, *p* < 0.05).

Figure 3 illustrates the antioxidant activity of EHE based on the concentration of malondialdehyde (MDA) formed by the lipid peroxidation induced by AAPH (A) and FeSO_4_ (B).

The extract was able to reduce the lipid peroxidation induced by AAPH, as indicated by a lower concentration of MDA compared with the white used, and was similar to the Trolox standard, which reduced the formation of MDA (product of lipoperoxidation) by approximately 55%. EHE exhibited an antioxidant effect presenting as an activity statistically equivalent to the standard used (Trolox, SL, USA). The difference between the results for AAPH and FeSO_4_ can probably be attributed to the affinity of the secondary metabolites of each fraction to the type of free radicals formed by each inducer.

**Figure 4:**
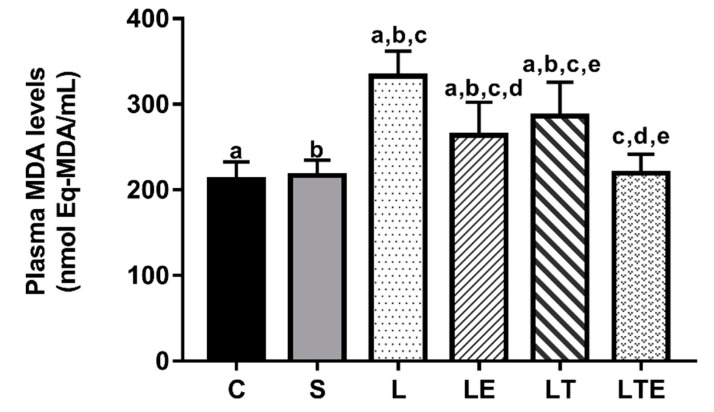
Plasma malondialdehyde (MDA) analysis. Plasma malondialdehyde (MDA) levels (nmol eqMDA/mL) after interventions in the different groups: C, control group; S, sham group; L, injury group; LE, injury + extract group; LT, injury + training group; LTE, injury + training + extract group. Values are expressed as mean ± standard deviation (*n* = 10 in each group). The letters “a”, “b”, “c”, “d” and “e” when repeated indicate statistical differences between the groups as determined by one-way ANOVA with Bonferroni post-hoc correction.

Figure 4 shows the plasma MDA levels. Statistically significant differences were found between the following groups: C (212 ± 20.5 nmol Eq-MDA/mL; 95% CI, 198–227) and L (333 ± 29.5 nmol eqMDA/mL; 312–354; *p* < 0.0001), and C and LE (264 ± 38.6 nmol eqMDA/mL; 236–291; *p* = 0.0038). Significant differences were also observed between the following groups: C and LT (286 ± 39.9 nmol eqMDA/mL; 258–315; *p* < 0.0001), S (217 ± 18 nmol eqMDA/mL; 204–230) and L (*p* < 0.0001), S and LE (*p* = 0.0115), S and LT (*p* < 0.0001), L and LE (*p* < 0.0001), L and LT (*p* = 0.0123), L and LTE (219 ± 22.3 nmol eqMDA/mL; 204–235; *p* < 0.0001), LE and LTE (*p* = 0.0210), and LT and LTE (*p* < 0.0001).

**Figure 5:**
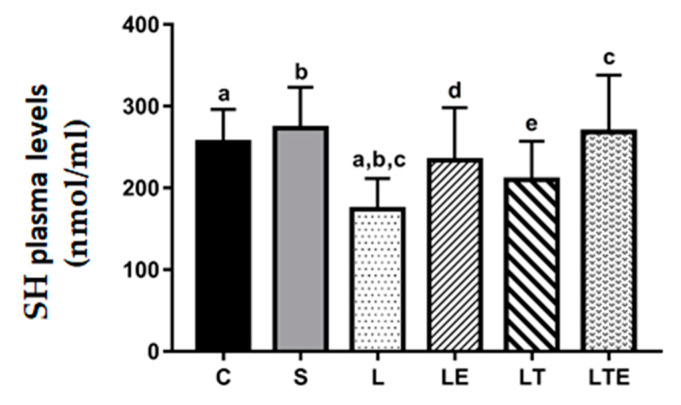
Plasma sulfhydryl (SH) analysis. Plasma sulfhydryl (SH) levels (nmol/mL) after interventions in the different groups: C, control group; S, sham group; L, injury group; LE, injury + extract group; LT, injury + training group; LTE, injury + training + extract group. Values are expressed as mean ± standard deviation (*n* = 10 in each group). The letters “a”, “b”, “c”, “d” and “e” when repeated indicate statistical differences between the groups as determined by one-way ANOVA followed by Bonferroni’s post-hoc correction.

Regarding plasma SH levels, significant differences were observed between the following groups: C (256 ± 40.4 nmol/mL; 95% CI, 225–287) and L (174 ± 37.7 nmol/mL; 143–203; *p* = 0.0275), C and S (273 ± 50 nmol/mL; 235–312; *p* = 0.0034), and L and LTE (268 ± 69.7 nmol/mL; 212–322; *p* = 0.0062) (Figure 5).

**Figure 6:**
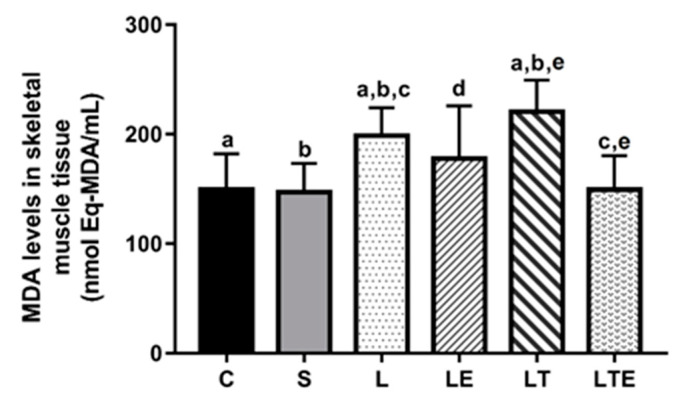
Assessment of MDA levels in the right gastrocnemius muscle. Malondialdehyde (MDA) levels (nmolEq-MDA/mL) in the gastrocnemius muscle after interventions in the different groups: C, control group; S, sham group; L, injury group; LE, injury + extract group; LT, injury + training group; LTE, injury + training + extract group. Values are expressed as mean ± standard deviation (*n* = 10 in each group). The letters “a”, “b”, “c”, “d” and “e” when repeated indicate statistical differences between the groups as determined by one-way ANOVA with Bonferroni post-hoc correction.

Regarding MDA levels in the gastrocnemius muscle, statistically significant differences were observed between the following groups: C (150 ± 32.4 nmol eqMDA/mL; 95% CI, 127–173) and L (199 ± 25.4 nmol eqMDA/mL; 128–166; *p* = 0.0234),C and LT (221 ± 28.7 nmol eqMDA/mL; 241–200; *p* = 0.002); S and L (147 ± 26.4 nmol eqMDA/mL; 166–128; *p* = 0.0134), S and LT (*p* < 0.0001), L and LTE (149 ± 30.9 nmol eqMDA/mL; 200–241; *p* = 0.0221), and LT and LTE (*p* = 0.002) (Figure 6).

**Figure 7:**
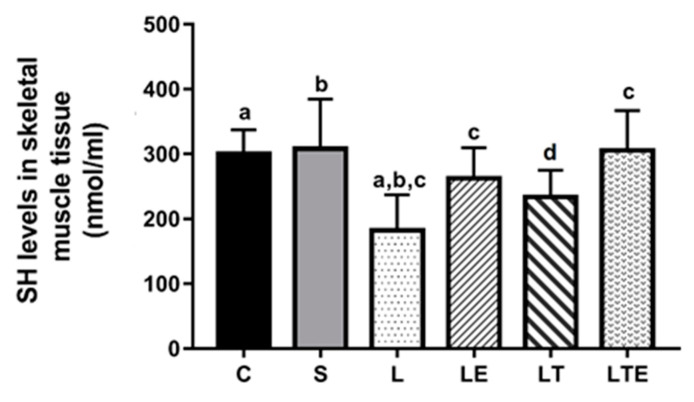
Evaluation of SH levels in the right gastrocnemius muscle. Sulfhydryl levels in the gastrocnemius muscle (nmol/mL) after interventions in the different groups: C, control group; S, sham group; L, injury group; LE, injury + extract group; LT, injury + training group; LTE, injury + training + extract group. Equal The letters “a”, “b”, “c” and “d” when repeated indicate statistical differences between the groups as determined by one-way ANOVA with Bonferroni *post-hoc* correction.

Regarding SH levels in the gastrocnemius muscle, statistically significant differences were observed between the following groups: C (301 ± 36.5 nmol/mL; 95% CI, 274–327) and L (182 ± 46.8 nmol/mL; 229–296; *p* = 0.0004), S and L (308 ± 76.7 nmol/mL; 253–263; *p* = 0.0002), L and LE (263 ± 46.8 nmol/mL; 229–296; *p* = 0.0444), and L and LTE (306 ± 60.9 nmol/mL; 262–349) (Figure 7).

**Figure 8:**
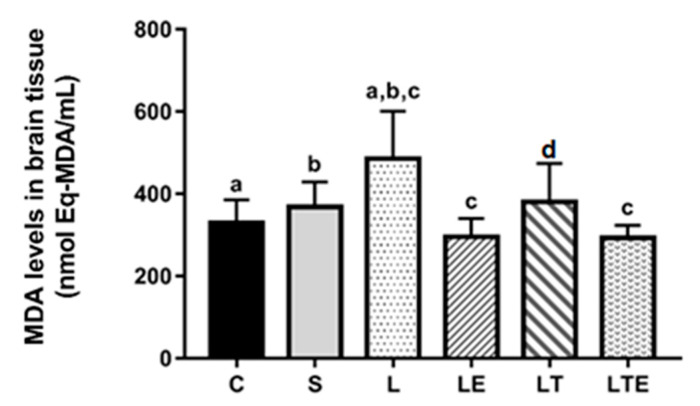
Analysis of MDA levels in the brain. Brain malondialdehyde levels (nmolEq-MDA/mL) after interventions in the different groups: C, control group; S, sham group; L, injury group; LE, injury + extract group; LT, injury + training group; LTE, injury + training + extract group. Values are expressed as mean ± standard deviation (*n* = 10 in each group). The letters “a”, “b”, “c” and “d” when repeated indicate statistical differences between the groups as determined by one-way ANOVA with Bonferroni post-hoc correction.

Regarding MDA levels in the brain, significant differences were observed between the following groups: C (330 ± 55.1 nmol eqMDA/mL; 95% CI, 288–372) and L (486 ± 116 nmol eqMDA/mL; 397–575; *p* = 0.0006), S and L (368 ± 60.6 nmol eqMDA/mL; 322–415; p = 0.0191), L and LE (295 ± 45.1 nmol eqMDA/mL; 261–330; *p* < 0.0001), and L and LTE (293 ± 30 nmol eqMDA/mL; 270–316; *p* < 0.0001) (Figure 8).

**Figure 9:**
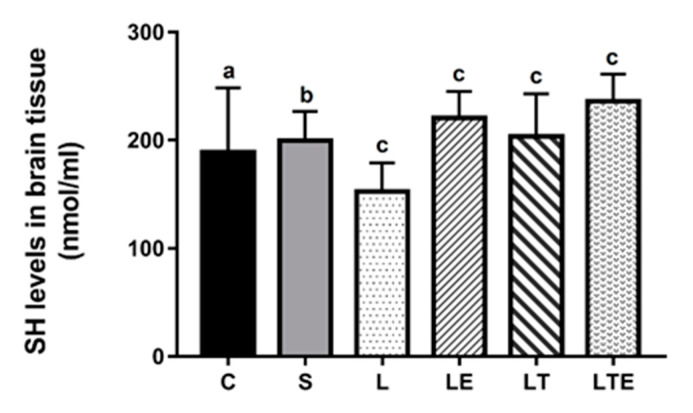
Evaluation of SH levels in the brain. Brain sulfhydryl levels (nmol/mL) after interventions in the different groups: C, control group; S, sham group; L, injury group; LE, injury + extract group; LT, injury + training group; LTE, injury + training + extract group. Values are expressed as mean ± standard deviation (*n* = 10 in each group). The letters “a”, “b” and “c” when repeated indicate statistical differences between the groups as determined by one-way ANOVA with Bonferroni post-hoc corrections.

Regarding brain SH levels, significant differences were observed between the following groups: L (153 ± 26.5 nmol/mL; 95% CI, 134–172) and LE (221 ± 24.4 nmol/mL; 203–238; *p* = 0.0013), L and LT (204 ± 39.3 nmol/mL; 176–232; *p* = 0.0370), and L and LTE (236 ± 25.1 nmol/mL; 218–254; *p* < 0.0001) (Figure 9).

**Figure 10:**
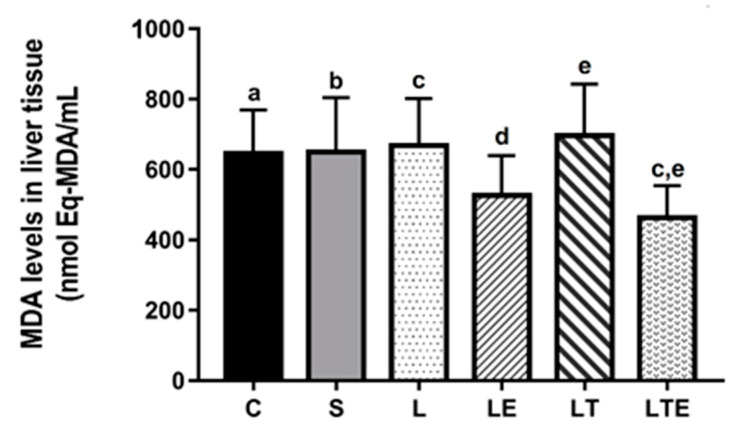
Analysis of MDA levelsin the liver. Malondialdehyde levels (nmolEq-MDA/mL) in the liver after interventions in the different groups: C, control group; S, sham group; L, injury group; LE, injury + extract group; LT, injury + training group; LTE, injury + training + extract group. Values are expressed as mean ± standard deviation (*n* = 10 in each group). The letters “a”, “b”, “c”, “d” and “e” when repeated indicate statistical differences between the groups as determined by one-way ANOVA with Bonferroni post-hoc correction.

Regarding liver MDA levels, statistically significant differences were observed between the following groups: L (668 ± 135 nmol eqMDA/mL; 95% CI, 564–771) and LTE (463 ± 91.7 nmol eqMDA/mL; 3933–544; *p* = 0.0230) and LTE and LT (696 ± 146 nmol eqMDA/mL; 809–584; *p* = 0.055) (Figure 10).

**Figure 11:**
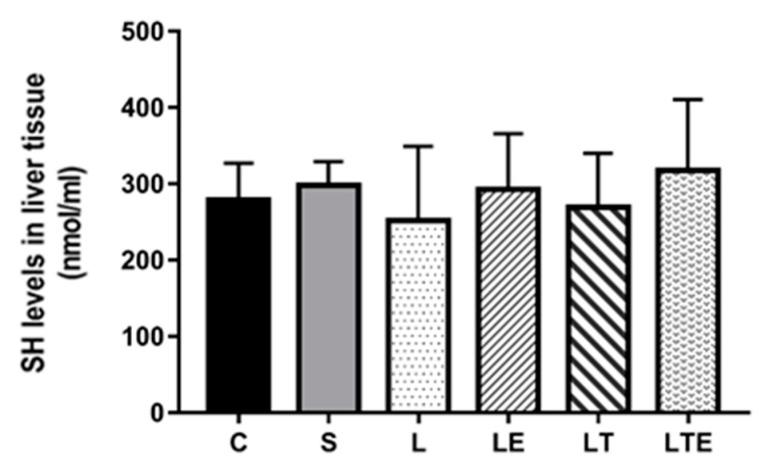
Assessment of SH levels in liver tissue. Sulfhydryl levels (nmol/mL) in the liver after intervention in the different groups: C, control group; S, sham group; L, injury group; LE, injury + extract group; LT, injury + training group; LTE, injury + training + extract group. Values are expressed as mean ± standard deviation (*n* = 10 in each group).

Regarding hepatic SH levels, we found no significant differences between the groups (Figure 11).

## 4. Discussion

We analyzed the indicators of OS and muscle damage in PNI rats subjected to RT sessions and EHE administration. We found that RT associated with oral EHE promoted a decrease in the levels of the OS markers.

OS is involved in several deleterious processes in PNI. Reactive species can react with most biological macromolecules, resulting in their oxidative modification; this results in the loss of their function [36]. In addition, reactive species are responsible for inducing an increase in the levels of inflammatory mediators at the injury site, where they activate glial cells, which are responsible for amplifying the inflammatory process by synthesizing and releasing pro inflammatory mediators, thereby promoting a pro inflammatory cascade [37].

Skeletal muscle function is closely associated with motor neuron innervation [38]. By interrupting neuromuscular communication, PNI induces skeletal muscle damage, triggering a decrease in muscle fibers because the muscle no longer receives the contraction signals necessary for its function [39]. Therefore, the inhibition of OS in PNI can accelerate the repair process and improve functional recovery after nerve injury. If OS persists and is not effectively suppressed in a timely manner, functional recovery is affected [40].

The results revealed that PNI induction caused a significant increase in the plasma, right gastrocnemius muscle (innervated by the injured nerve), and brain MDA levels after 21 days of surgery. MDA is the primary biomarker of oxidative damage [41]. This biomarker consists of the final product of a series of chemical reactions that occur in lipid peroxidation, in which, basically, it would be the degradation of the cell membrane by the action of reactive species, being measured by the formation of MDA through its complex action with thiobarbituric acid [42].

The increase in reactive species is considered a major source of neural damage after PNI and plays a negative role in functional nerve recovery after injury [40]. Uslusoy et al. [43] demonstrated an increase in plasma, skeletal muscle, and brain MDA levels after 28 days of sciatic nerve crushing in Wistar rats. The changes were because of high oxygen consumption, iron content, and polyunsaturated fatty acid levels and low antioxidant levels in these organs.

In the present study, we observed that RT promoted an increase in MDA locally in the skeletal muscle. However, it may have caused a systemic antioxidant effect considering the fact that plasma MDA levels were lower in group L than in group LT. Chen et al. [44] and Chris et al. [45] performed treadmill exercise protocols in rats with streptozotocin-induced diabetic PNI and observed exercise-induced decrease in MDA levels after five weeks and 27 days, respectively.

In addition, the present study demonstrated that the group that received only EHE also demonstrated a decrease in blood and muscle MDA levels, similar to groups C and S (without nerve damage). Concomitantly, injured rats that were submitted to RT associated with oral EHE demonstrated results similar to the groups without PNI, both in the blood and skeletal muscle. This finding corroborates with the results of the study by dos Santos et al. [27], which demonstrated that a combination of RT and oral EHE in Wistar rats promotes a greater antioxidant effect than EHE alone.

Ischemic processes during RT lead to the generation of reactive oxygen species (ROS) [46]. The moderate increase in ROS in the skeletal muscle favors redox signaling, which promotes benefits to the muscle cell, such as strength production during contraction, glucose uptake, and increase in exogenous antioxidants throughout the body [47]. Such responses are dependent on the type, duration, and intensity of exercise [48]. The increase in antioxidants occurs through the activation of factor 2 related to the erythroid nuclear factor 2 (NRF_2_), a powerful nuclear transcription factor that coordinates the antioxidant cytoprotective system, which plays an important role in preventing the oxidation of molecules [49].

With respect to the studies in humans, it has been observed that various RT protocols promote reduction in oxidative damage in several groups. Park and Kwak [50] found that RT did not increase serum MDA levels after exercise in male athletes, whereas untrained individuals showed a substantial increase in serum MDA levels. Nemati et al. [51] concluded that women also produce less redox imbalance caused by continuous RT sessions. Regarding the elderly, Padilha et al. [52] observed that a 12-week RT with a frequency of two days a week improved muscle strength and OS in people over 60 years.

Since the last few years, studies using natural products have shown positive results as a therapy in PNI. Zhao et al. [53] have demonstrated that the *Radix puerariae Lobatae* extract promotes greater expression of neurotrophic factors and the activation of protein synthesis pathways in PNI. In addition, the red propolis extract promotes regenerative responses and accelerates functional recovery after sciatic nerve crush [54]. Furthermore, sesame oil promotes better functional recovery after LNP induction [55]. These results are mainly attributed to the antioxidant and anti-inflammatory properties of the compounds used [54].

The effects of *B. virgilioides* have been described, and it has scientifically been proven to have a modulating effect on T lymphocytes [56], antibacterial action [57], anti-inflammatory effect [58], analgesic effect [59], antinociceptive effect [60], anxiolytic effect [61], and antioxidant effect [27]. The EHE of the bark of *B. virgioloides* demonstrates high antioxidant action both in vitro and in vivo. It features a fingerprint characteristic of medium-to-high polar substances, similar to phenolic compounds [27]. Phenolic compounds are chemical structures made up of one or more aromatic rings with hydroxyl moieties. They protect lipids, proteins, carbohydrates, and DNA from oxidative damage by reducing free radicals, making them stable after donating an electron from the hydroxyl to the radical. In addition, phenolic compounds act to increase the levels of exogenous antioxidants, such as the enzymes glutathione peroxidase, ascorbic acid, and superoxide dismutase [62,63].

In addition to OS in the organ innervated by the injured nerve, PNI promotes the release of certain inflammatory mediators and ERs into the plasma and the dorsal root ganglia, which in turn ascend to the brain. Surprisingly, little attention is paid to the central nervous response [64]. OS in neural tissues causes signaling and interrupted homeostasis and is responsible for numerous events ranging from the protein catabolism to neuronal death. Higher levels of reactive species expression also activate nuclear factor kappa B, which in turn initiates the transcription of pro-inflammatory mediators in the central nervous system [64,65].

In the present study, we observed that there was an increase in MDA levels caused by PNI. The LT and LTE groups demonstrated a significant decrease in brain MDA levels compared with the L group. Accumulating evidence suggests the possible beneficial effects of polyphenols on neuronal protection via OS circumvention. Polyphenols can cross the blood–brain barrier by modulating the central nervous system [66]. Uslusoy et al. [43] demonstrated that the administration of the extract of *Hypericum perforatum* L., a medicinal plant that contains polyphenols within its active compounds, reduced brain MDA levels after 28 days of PNI induction in Wistar rats.

In addition, in the present study, MDA levels in the liver were assessed. Peripheral nerve damage did not increase MDA level in the organ. However, the groups administered EHE did not demonstrate an increase in hepatic MDA, which if increased would be an important indicator of the hepatotoxic effect caused by the extract.

Moreover, plasma SH levels were significantly lower in group L than in groups C, S, and LTE, demonstrating similar results between them. In the right gastrocnemius muscle, SH level was higher in groups C, S, LE, and LTE, and the levels in the trained group were comparable to those in the groups without injury. SH consists of an indirect antioxidant defense biomarker and is found in the GSH Cys residue and other antioxidants [67]. The molecules contained in the SH side chain group act as antioxidants, stabilizing free radicals when receiving their unpaired electron [68]. When assessed in the brain, the three groups with injury submitted to therapy demonstrated higher levels of SH than group L.

Uslusoy et al. [43] demonstrated that the administration of *H. perforatum* L. for 28 days, which has phenolic compounds as an active ingredient, caused an increase in antioxidant substances (GSH, melatonin, GPX, and retinol) in the brain, blood, and skeletal muscle of Wistar rats submitted to sciatic nerve compression. Safakhah et al. [11] demonstrated that exercise on a treadmill for 3 weeks promotes an increase in the total antioxidant capacity in the serum of Wistar rats submitted to PNI. The changes in relation to the SH levels observed in the present research caused by exercise and by the use of HE and both reinforce the idea that the therapies used have a positive action in the control of PNI-induced OS.

## 5. Conclusions

In conclusion, PNI promotes an increase in OS markers, which, in turn, are related to a decrease in skeletal muscle mass and motor function. The combined treatment with RT and *B. virgilioides*–derived EHE reduces plasma, gastrocnemius muscle, and brain MDA levels and simultaneously causes an increase in SH levels in the same organs after PNI induction in Wistar rats. Therefore, the use of EHE and RT has the potential to reduce nerve-injury-induced OS.

## Figures and Tables

**Table 1:** Phytochemical prospecting of the hydroethanolic extract (EHE) of *Bowdichia virgilioides*.

Components	EHE
Phenols	+
Tannins	+
Flavonoids	+
Xanthones	+
Categories	+
Pentacyclic triterpenoids and free steroids	+
Saponins	−
Alkaloids	+

**Table 2:** Antioxidant activity of the hydroethanolic extract and fractions of *B. virgilioides* as determined by reducing the free radical 2,2-diphenyl-1-picrylhydrazyl (DPPH^•^).

Sample	EC_50_(µg/mL)	PI (%) *	AAI **
EHE	29.50 ± 2.40 ^a^	42.90	0.84
GallicAcid	1.05 ± 0.20 ^a^	92.06	23.80

* Percentage of inhibition (PI) of free radical formation. ** Antioxidant activity index (AAI). EC_50_ results are expressed as mean and standard deviation. The letter ^“a”^ when repeated indicate statistical difference between the groups (*n* = 3; ANOVA followed by Tukey’s test, *p* < 0.05). EHE = hydroethanolic extract.
